# Non-Destruction Evaluation Method for Long-Term Oxidation Behavior of SiC/SiC Composites with Different Preforms in Wet Oxygen

**DOI:** 10.3390/ma15113812

**Published:** 2022-05-27

**Authors:** Guodong Sun, Yan Wu, Xin’gang Luan, Xinming Xu, Xinxin Cao, Jiahao Zhang, Huan Zhao, Qing Zhang, Laifei Cheng

**Affiliations:** 1 School of Materials Science and Engineering, Chang’an University, Xi’an 710064, China; wyyan15779091838@163.com (Y.W.); zh18292001780@163.com (H.Z.); 2 Science and Technology on Thermostructural Composite Materials Laboratory, Northwestern Polytechnical University, Xi’an 710072, China; xuxinming@mail.nwpu.deu.cn (X.X.); cwenyuan163@163.com (X.C.); 17866639312@163.com (J.Z.); zhangqing@nwpu.edu.cn (Q.Z.); chenglf@nwpu.edu.cn (L.C.)

**Keywords:** SiC/SiC composites, wet oxygen environment, non-destructive testing, oxidation evaluation

## Abstract

The implementation of SiC fiber reinforced SiC/SiC composites to aero-engine hot components has attracted wide attention, due to their many excellent properties. Along these lines, in order to predict the oxidation behavior of the material in extreme environments and to explore the effect of different preforms on the oxidative behavior of the composites, four SiC/SiC composites, with different preforms, were oxidized under environmental conditions of pressure of 12 kPa H_2_O:8 kPa O_2_:80 kPa Ar, at 1400 °C temperature. Moreover, the morphology and defect distribution of the samples were characterized by carrying out scanning electron microscopy, and micro-computed X-ray tomography measurements. Furthermore, the relation between the micro- and macro-scales was established, so as to be able to predict the oxidation behavior of the composites; not only the quantitative relationship between the mass change rate and the defect volume change rate, but also the combination of micro-computed X-ray images.

## 1. Introduction

The utilization of silicon carbide fiber for reinforcing silicon carbide composites (SiC/SiC) is considered of great importance, since these composites are some of the most potentially used engine thermal structure materials, due to their advantages of being lightweight, and having good mechanical properties and robust oxidation resistance [1,2,3]. On top of that, some SiC/SiC components have already been used in the LEAP aero-engine by General Electric Company (GE) [4,5,6]. This remarkable achievement was attributed to the implementation of intensive research that was carried out in different environments. As a result, those environmental tests built up a better understanding of the change in the micro- and macro-behaviors of SiC/SiC composites and were beneficial for the improvement of the composites.

For example, Nasiri et al. [7] explored the oxidation behavior of SiC/SiC composites in air at 1200–1400 °C. In another interesting work, Mohan et al. [8] conducted both a static air (1200–1400 °C, 100 h) and an oxygen-acetylene combustion test on SiC/SiC (β-SiC coating) composite material that was prepared by the isothermal chemical vapor infiltration (ICVI) method. Additionally, Zhao et al. [9] performed air oxidation at 800–1200 °C for 100 h on the SiC/SiC-SiBC composite, and performed related mechanical tests on the oxidized samples. In the report of Jiang et al. [10], 3D SiC/SiC composites, with different interfaces, were placed under static oxidation at 1100 °C for 400 h, and their flexural properties and fracture modes were compared. Sun et al. [11] compared two different SiC/SiC-SiBC composite materials against static air oxidation at 800, 1000 and 1200 °C for 100 h. Luan et al. [12,13] studied different SiC/SiC-SiBCN composite materials at different temperatures (1200–1400 °C), their long-term static oxidation in different atmospheres, and their creep behaviors under certain environments.

In the above-mentioned studies, scanning electron microscopy (SEM) and transmission electron microscopy (TEM) were actively used to deeply characterize the fracture cross-sections of the samples. Consequently, the relationship between the micro-structure and the oxidation behavior that changed with temperature, time and environment was explored. However, the relationship that originates from destructive testing procedures cannot support real-time health monitoring of the SiC/SiC components during real applications. Therefore, it is necessary to conduct non-destructive testing routines to closely examine the changes of the oxidation behavior of each component during the oxidation process.

At present, the micro-computed X-ray tomography (μ-CT) test [14,15,16,17] is widely used for non-destructive testing of the internal microstructure of composite materials to obtain information about change in the microstructure. For instance, Frazer et al. [18,19] employed the nanoindentation technique, Raman spectroscopy and μ-CT to obtain the difference in the mechanical properties between the fiber and matrix of the SiC/SiC configuration. However, the relationship between the micro- and the macro-properties has not yet been constructed.

With this in mind, this work considers aeroengines as the application background, and a wet-oxygen atmosphere was selected as the impact condition for studying four kinds of SiC/SiC composites with different preforms. In addition, routine macro tests (weight change, flexural strength) and micro tests (SEMs) of the materials were adopted. Moreover, μ-CT technology was used to conduct non-destructive testing, and to obtain the defect distribution of the samples after enforcing different oxidation times, so as to better explain the oxidation processes of the materials. Interestingly, after integrating the obtained data, the influence of the preform on the changes of both macro-property and micro-property was explained, and a function relating to the macro-property and micro-property was further constructed. The extracted results not only provide a deep understanding of the relationship between the microstructure and the oxidation behavior of the SiC/SiC compound, but also predict its macroscopic properties at different times to provide real-time health monitoring of the SiC/SiC components.

## 2. Experiment

### 2.1. Materials

Table 1 shows the main physical properties of the SiC fibers used in this work (from Fujian Leadasia New Material Co., Ltd., Quanzhou, China). The gas sources that were used in the employed oxidation experiment were argon with a purity of 99.99% and oxygen with a purity of 99.99% (from Xi’an Weiguang Gas Co., Ltd., Xi’an, China). Furthermore, deionized water, with a conductivity value of less than 0.5 μS/cm (Xi’an Xinyuan Distilled Water Plant, Xi’an, China), was also used in this study.

### 2.2. Preparation of the SiC/SiC Samples

Four different types of SiC fiber preforms were all braided by Xinyao Company: more specifically, a two-dimensional plain weaving preform, a satin weaving preform and a three-dimensional four-directional preform were denoted as 2D plain, 2D satin and 3D 4d, respectively. A two-dimensional plain/satin weaving preform was produced by laminating plain weaving cloth and satin weaving cloth in a ratio of 1:1 and was denoted as 2D P/S. The chemical vapor infiltration (CVI) method was used to prepare both the BN interface and the SiC matrix, due to its comparative advantages of low thermal and mechanical stress during the densification process [20,21]. After depositing the SiC matrix six times, the obtained SiC/SiC plate was cut into samples with a size of 40 × 5 × 3 mm^3^. Then, several deposition times of the SiC were carried out until the open porosity of the samples reached the value of 9%.

### 2.3. Oxidation Tests

A GSL 1600X tube furnace (produced by Hefei Kejing, Hefei, China) was used to perform the oxidation experiment. The specific test process is described as follows. When the temperature of the tube furnace reached 1400 °C, an oxidizing atmosphere (12 KPa H_2_O:8 KPa O_2_:80 KPa Ar) was introduced, and then the SiC/SiC composite materials were pushed into the center of the constant temperature zone of the tube furnace eight times to start oxidation. Each time stay was for at least 5 min. After being oxidized for 10, 30, 50, 70, 100, 150, 200, 250 and 300 h, respectively, the samples were taken out to be weighed with an analytical balance (Mettler Toledo, AG 204, Zurich, Switzerland). During the whole process, the samples did not heat up and cool down with the furnace.

### 2.4. Mechanical Tests

A three-point bending test with a span of 30 mm and a loading rate of 0.5 mm/min was performed on the SiC/SiC compound with different oxidation times (100, 200 and 300 h) at room temperature. The equipment that was used was an electric loading frame (CMT 4304, Sans Materials Testing Co., Ltd., Shenzhen, China), while four samples were selected for each test.

### 2.5. Microstructural Characterization Tests

The fracture cross-section morphology of the SiC/SiC configuration was observed by using SEM (S-4700, Hitachi, Chiyoda, Japan) imaging. The distribution of the internal defects was measured by μ-CT (YXLON Y. Cheetah, Hamburg, Germany), whereas the volume fraction of the defects within the samples was calculated by using VG Studio MAX 3.0.2 software.

## 3. Results and Discussion

### 3.1. Original SiC/SiC Composite Structure

**Figure 1:**
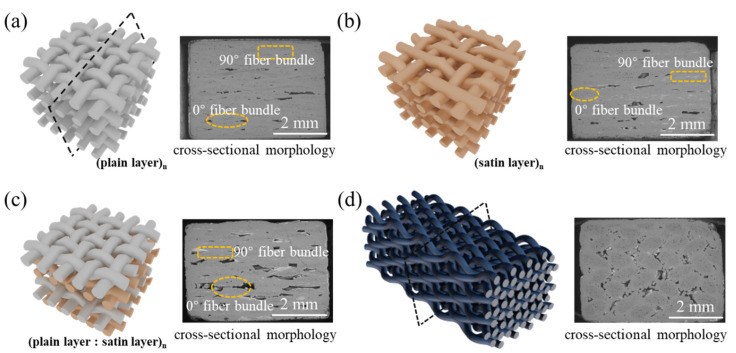
Structure diagram and cross-sectional morphology of four SiC/SiC composite materials (**a**) 2D plain; (**b**) 2D satin; (**c**) 2D P/S; (**d**) 3D 4d.

Figure 1 demonstrates the structure diagrams and cross-sectional morphologies of the original composite materials, which were combined for analysis. More specifically, Figure 1a shows the 2D plain weave structure, wherein fiber bundles (divided into warp yarns and weft yarns) composed the SiC fibers, interwoven in a plane according to the rule of one up and down to form a fiber cloth layer. Then, according to the thickness of the required material, the corresponding number of fiber cloth layers (n) was selected and laid to form a preform with a plain weave structure. The characteristic of this structure is that there are many weaving sites between the fiber bundles, and there are also more “constraints” between them. Figure 1b exhibits the 2D five-piece satin structure. Due to having fewer weaving sites between the fiber bundles during the enforcement of the weaving process, this structure has a large volume of defect holes. Its morphology can be used to more clearly understand the difference in the internal structures of a 2D plain weave and 2D satin weave, owing to the different weaving methods. According to μ-CT imaging technology, the results of each 20 μm slice confirmed that the P/S structure (as shown in Figure 1c) was obtained by laminating both the plain weave cloth layer and the satin cloth layer in a ratio of 1:1. This structure also has two structural characteristics of 2D plain and 2D satin in the XY direction. It can be seen from the cross-sectional morphology that the 2D P/S composite material has large volume defect pores. The 3D 4d preform, with an integral characteristic, was obtained by moving the fiber bundle periodically through the yarn carrier according to a certain rule that is displayed in Figure 1d. The mechanical properties in the Z-direction of the 3D 4d are better than the two-dimensional materials, and there is no delamination in the process of mechanical testing, due to the integrity of this structure.

### 3.2. Oxidation Behavior of the Different Kinds of SiC/SiC

**Figure 2:**
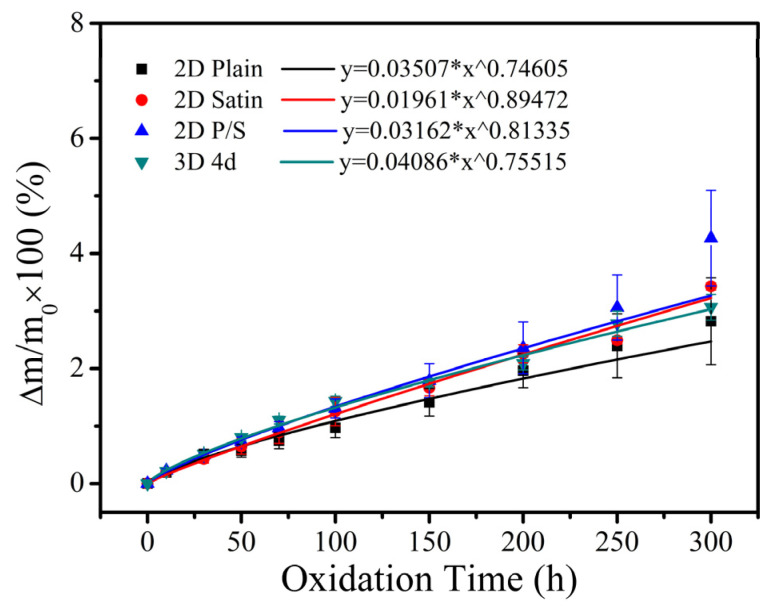
Fitting curves of mass change of four kinds of SiC/SiC composites after being oxidized in 12 H_2_O:8 O_2_:80 Ar.

Figure 2 illustrates the mass change curves of four kinds of SiC/SiC composites after being oxidized at 1400 °C in wet oxygen for 300 h. As can be ascertained from the acquired outcomes, all the SiC/SiC composites exhibited a continuous weight gain during the oxidation test, indicating that the weight gain, caused by the formation of oxides (SiO_2_), was higher than the respective weight loss, induced by the release of the gas phase products (Si(OH)_4_, B_2_O_3_). Moreover, the weight gain rate of the 2D Plain, 2D Satin, 2D P/S and 3D 4d composites was 2.82%, 3.43%, 4.27% and 3.07%, respectively. As far as the difference in changes was concerned, a detailed analysis was carried out in conjunction with the extracted results of the μ-CT that will be presented in the next section. Furthermore, all four composites exhibited parabolic growth from the non-linear fitting curves of the mass change, suggesting that the oxidation process of materials in wet oxygen at 1400 °C is controlled by a diffusion-based mechanism, while the oxidation rate of the composites gradually slows down over time. It is also interesting to notice that, as the oxidation progresses, the oxide layer formed on the surface and the defects of the sample gradually became thicker, resulting in a slow decrease in the rate of oxygen and water vapor molecules entering the sample, and consequently slowing down of the mass increase of the composite material. For SiC/SiC composites, due to the dispersion of defects in the material itself, the channel and speed of the diffusion of the oxidizing atmosphere into the sample will be affected during the oxidation process. Therefore, even for the same composite material, under the same oxidative environment, there will be a certain difference in mass change, and there will be a standard deviation at each measurement time point, but it will not affect the experimental results.

**Figure 3:**
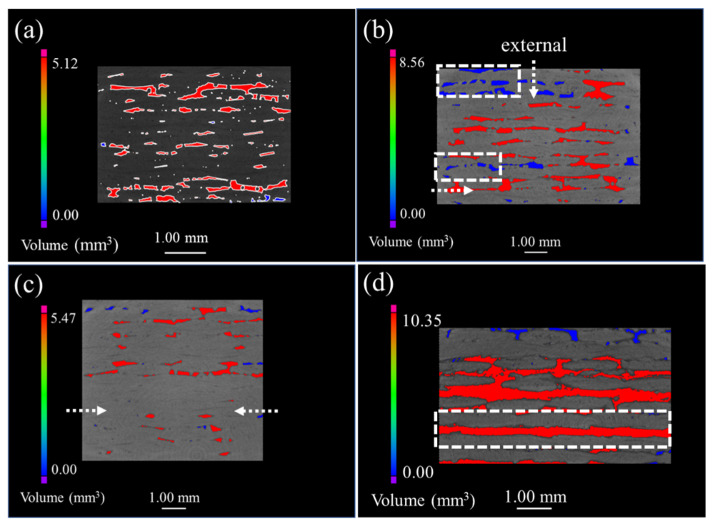
μ-CT images of 2D plain SiC/SiC composites with different oxidation times in 12 kPa H_2_O:8 kPa O_2_:80 kPa Ar at 1400 °C: (**a**) as fabricated; (**b**) 100 h; (**c**) 200 h; (**d**) 300 h.

**Figure 4:**
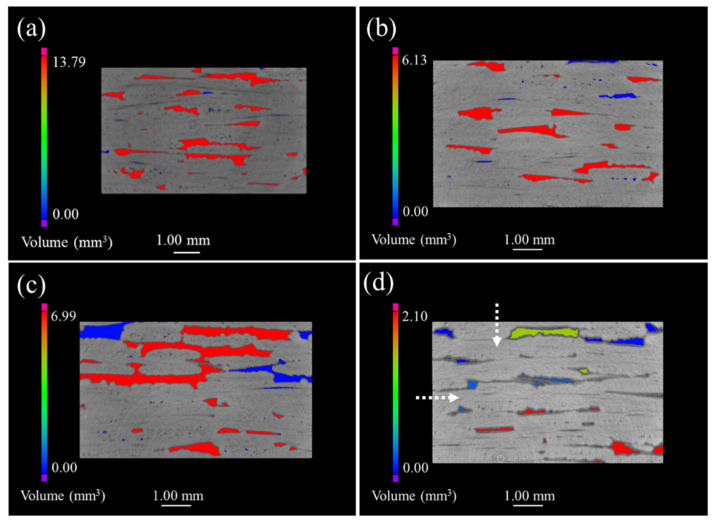
μ-CT images of 2D satin SiC/SiC composites with different oxidation times in 12 kPa H_2_O:8 kPa O_2_:80 kPa Ar at 1400 °C: (**a**) as fabricated; (**b**) 100 h; (**c**) 200 h; (**d**) 300 h.

**Figure 5:**
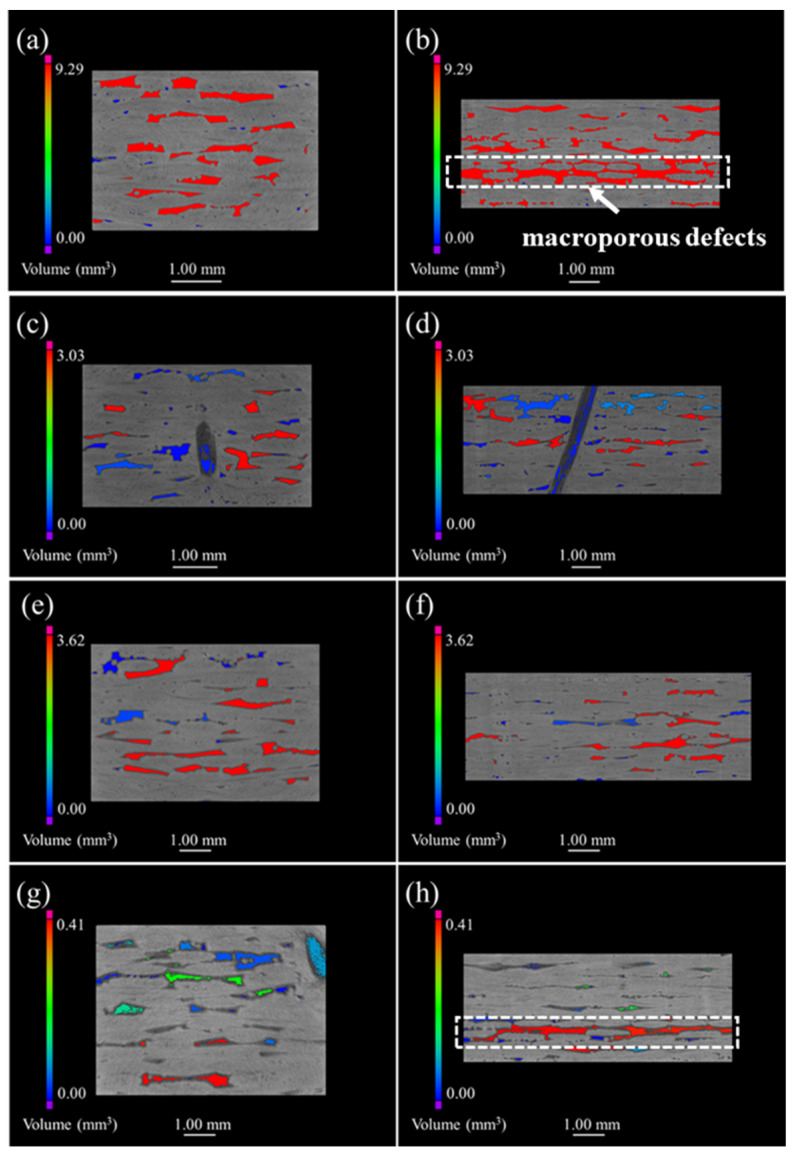
μ-CT images of 2D P/S SiC/SiC composites with different oxidation times in 12 kPa H_2_O:8 kPa O_2_:80 kPa Ar at 1400 °C: (**a**,**b**) as fabricated; (**c**,**d**) 100 h; (**e**,**f**) 200 h; (**g**,**h**) 300 h.

**Figure 6:**
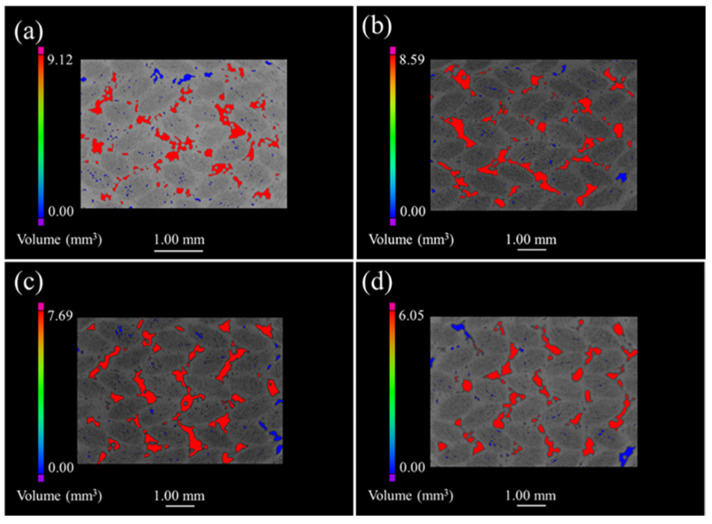
μ-CT images of 3D 4d SiC/SiC composites with different oxidation times in 12 kPa H_2_O:8 kPa O_2_:80 kPa Ar at 1400 °C: (**a**) as fabricated; (**b**) 100 h; (**c**) 200 h; (**d**) 300 h.

Figure 3, Figure 4, Figure 5 and Figure 6 demonstrate the μ-CT images of the SiC/SiC composites with four different preforms. The maximum defect volume change and defect distribution that are shown in all graphs are used to explain the oxidation process of the samples. Figure 3 represents the μ-CT images of both the original and the oxidized 2D plain SiC/SiC composites. The defects are mainly distributed in the inter-bundle and the inter-layer of the original SiC/SiC composites, as can be seen from Figure 3a. When the sample was oxidized for 100 h (as is shown in Figure 3b), many small-volume defects were distributed at the external surface of the sample and the connectivity of the macro-porous defects decreased. As a result, it is demonstrated that the oxidation of the composites proceeds from the external to the internal. However, the sample, after being oxidized for 300 h, had penetrating large-volume defects, which indicates that a delamination procedure occurs between the weave layers, while the generated oxides are accumulated in the woven holes at this time, forming a large stress concentration.

Figure 4 shows the μ-CT images of 2D satin SiC/SiC composites. It can be seen that the defects presented in Figure 4a are unevenly distributed and the volume is relatively large, compared with the 2D SiC/SiC composite. The maximum defect volume of composites after being oxidized for 100 h and 200 h was reduced by 50% and can be observed in Figure 4b,c. Simultaneously, a large number of small-volume defects appeared. Compared with the μ-CT morphology of the first 200 h, more defects of different volumes could be found (shown in Figure 4d), and the maximum defect volume reduced to 2.1 mm^3^. Therefore, we can argue that the oxides in the whole process had a good sealing effect on the sample, and no serious delamination phenomenon, similar to that of the 2D plain SiC/SiC composite material, took place.

The acquired μ-CT images of the 2D P/S composite are exhibited in Figure 5. More specifically, the defect volume and connectivity between the fiber layers were greater than those of the 2D plain and the satin composites, as observed in the μ-CT images of the as-fabricated 2D P/S SiC/SiC composite (in Figure 5a,b). When the sample was oxidized for 200 h, the maximum defect volume decreased by 67% compared with the original sample, indicating that the oxidation degree of the sample was relatively large. Moreover, the existence of different defect volumes, similar to the satin composite material, can be observed in Figure 5g,h, and the plain weave layer also appeared to have a similar defect volume to the penetrating defect of the 2D plain weave composite.

Figure 6 discloses the μ-CT images of the 3D 4d composite. The unoxidized samples are shown in Figure 6a where small pores and large defects are mainly and evenly distributed within the fiber bundle and the angle between the fiber bundle, respectively. Furthermore, Figure 6b–d illustrate that the defect volume decreased as oxidation progressed, while the reduction in the maximum pore volume of the oxidized sample, relative to the original sample, was 5.8%, 10.47% and 21.32%; which is small compared with the other three composites. In addition, the distribution of the defects in the oxidized composites was similar to the original samples, suggesting that the oxide filled the material defects more uniformly.

**Figure 7:**
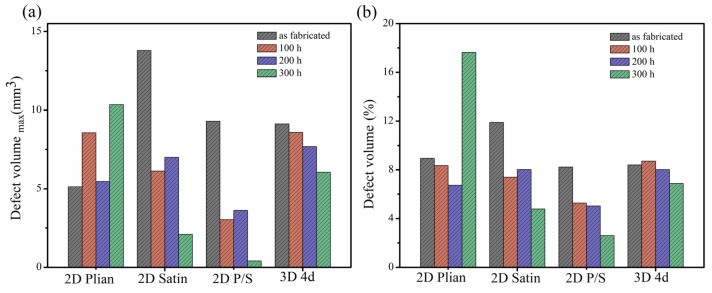
μ-CT results of four kinds of original and oxidized SiC/SiC composites: (**a**) Maximum pore size and (**b**) defect volume fraction.

Although the inter-bundle and the inter-layer defects were mainly distributed in the four kinds of composite materials, both the shape and size of the pores were different due to the difference in structures. For example, the defect holes of the 2D plain SiC/SiC configuration were smaller than those of the 2D satin weave, since the fiber bundles of the plain weave are tightly woven. Additionally, the 2D P/S composite materials experienced formation of large connectivity defects in the interlayer during the deposition of the matrix, because of the stacking of plain weave and satin weave. This effect can be considered to prove that the mass change rate of the 2D P/S composite is largely combined with the μ-CT images of the oxidized samples. However, the defects of the 3D 4d composites were mainly distributed at the angle between the fiber bundles uniformly since it is a woven material, and there was no interlayer defect like that of the two-dimensional material. Figure 7 displays the μ-CT data of four kinds of SiC/SiC composites. The defect volume of the unoxidized 2D satin composite was the largest (the change of the defect volume), as is shown in Figure 7a, whereas that of the 2D P/S composite, after being oxidized for 300 h, was the smallest. On top of that, the change of the defect volume fraction of the four kinds of SiC/SiC composites, represented in Figure 7b, and the overall change trend, was similar to the pore volume change.

**Figure 8:**
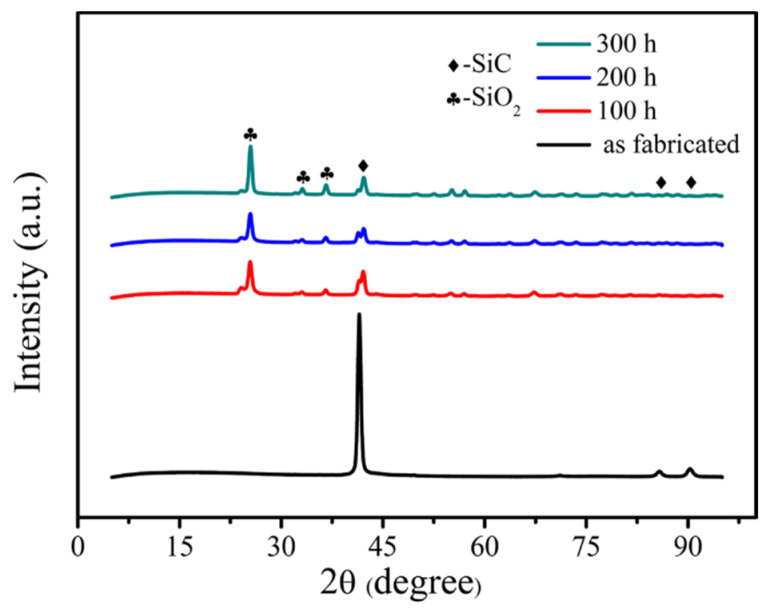
The results of X-ray diffraction of 2D satin SiC/SiC composites.

The samples were tested by X-ray diffraction to characterize the change of the phase composition of the material. The 2D satin SiC/SiC composite was selected as the representative, and its XRD results are shown in Figure 8. It can be seen that the original SiC/SiC composite is mainly composed of SiC phase. However, when the material was oxidized, the existence of SiO_2_ phase was detected on the surface of the sample, and the crystallinity of SiO_2_ gradually increased with increase in time. This indicates that the SiO_2_ film formed on the surface of the sample after oxidation, which can heal surface cracks and play a certain hindering effect on the oxidizing atmosphere. Additionally, the oxidizing atmosphere diffused from the outside to the inside, and SiO_2_ oxides were also generated inside to seal the defects, so that the volume fraction of internal defects decreased, as shown in Figure 7.

**Figure 9:**
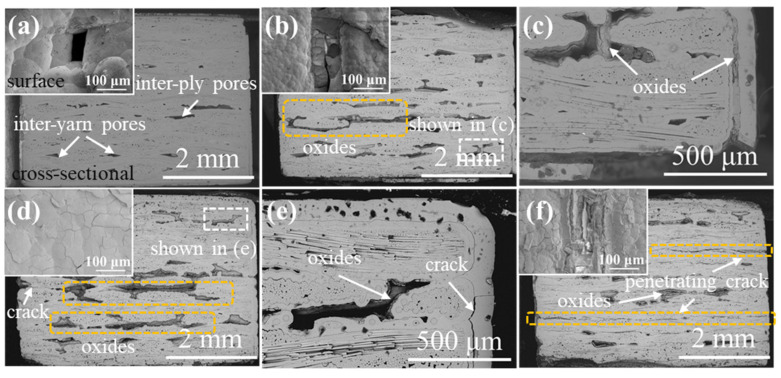
The cross-sectional morphology and surface morphology of 2D plain SiC/SiC composites (**a**) before and after being oxidized in 12 kPa H_2_O:8 kPa O_2_:80 kPa Ar at 1400 °C for (**b**,**c**) 100 h, (**d**,**e**) 200 h and (**f**) 300 h.

Figure 9 shows the cross-sectional morphology and surface morphology of the 2D plain SiC/SiC composites. From Figure 9a, it can be observed that there are obvious fiber bundle weaving holes on the surface of the original sample. It can be observed that the surface is filled with oxides (as shown in Figure 9b,d,f), and there is also the generation of microcracks. Moreover, obvious inter-bundle pores and inter-layer pores are exhibited in the cross-sectional morphology of the sample. Combined with the cross-sectional morphology of Figure 9b–f, it can be seen that a large number of oxides formed between the layers of the composite material. Furthermore, there were obvious penetrating cracks inside the material when oxidation was carried out for 300 h, which was consistent with the results in μ-CT images (as shown in Figure 2).

**Figure 10:**
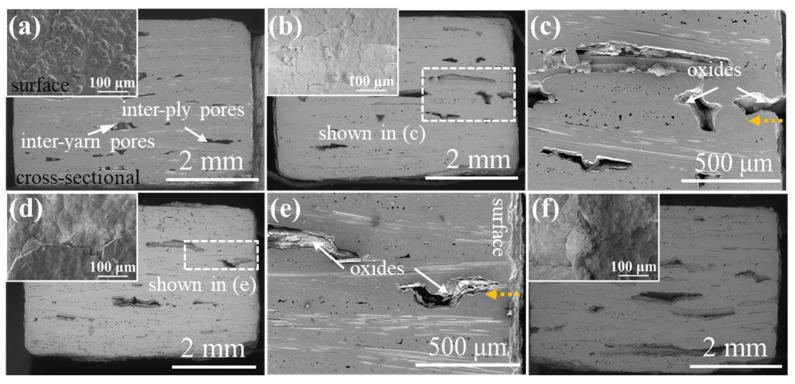
The cross-sectional morphology and surface morphology of 2D satin SiC/SiC composites (**a**) before and after being oxidized in 12 kPa H_2_O:8 kPa O_2_:80 kPa Ar at 1400 °C for (**b**,**c**) 100 h, (**d**,**e**) 200 h and (**f**) 300 h.

Figure 10 exhibits the cross-section morphology and surface morphology of the 2D satin SiC/SiC composite. From Figure 10a, it can be observed that the SiC on the surface of the original sample exhibits a cauliflower-like morphology, and oxides were gradually formed on the surface as oxidation progressed (combined with the XRD results in Figure 8). Observing the cross-sectional morphology in Figure 10a, the existence of inter-bundle pores and inter-layer pores can also be seen in the original sample, but the volume of defect pores is larger than that in the 2D plain SiC/SiC composite. Furthermore, it can be observed that the defects at the near surface (the position of the orange arrow in Figure 10c,e) are filled with a large amount of oxide, and the sealing degree of the sample after oxidation for 200 h was greater than that of oxidation for 100 h, indicating that oxidation proceeded inward along the interlayer. Furthermore, the defect pores near the surface of the sample oxidized for 300 h were well sealed, as shown in Figure 10f.

**Figure 11:**
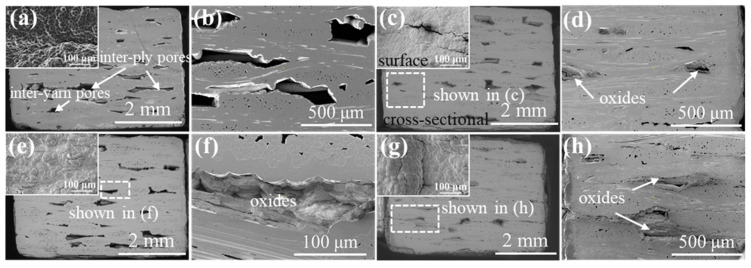
The cross-sectional morphology and surface morphology of 2D P/S SiC/SiC composites (**a**,**b**) before and after being oxidized in 12 kPa H_2_O:8 kPa O_2_:80 kPa Ar at 1400 °C for (**c**,**d**) 100 h, (**e**,**f**) 200 h and (**g**,**h**) 300 h.

Figure 11 displays the cross-section morphology and surface morphology of the 2D P/S SiC/SiC composite. Observing the surface morphology (as shown in Figure 11a,c,e,g), dense oxides were formed on the surface of the sample after oxidation. In the cross section of the original sample (in Figure 11a,b), it can be inspected that there are a large number of large-volume defect pores inside the 2D P/S composite, and the defect pores were filled with SiO_2_ after oxidation. In addition, it can be seen that a crack appeared between the inter-layers, shown in Figure 11g, and the defect pores near the surface of the sample were basically completely sealed (as shown in Figure 11h).

**Figure 12:**
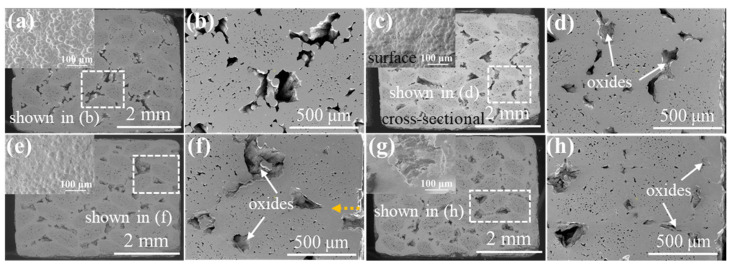
The cross-sectional morphology and surface morphology of 3D 4d SiC/SiC composites (**a**,**b**) before and after being oxidized in 12 kPa H_2_O:8 kPa O_2_:80 kPa Ar at 1400 °C for (**c**,**d**) 100 h, (**e**,**f**) 200 h and (**g**,**h**) 300 h.

As shown in Figure 12, it can be seen that there were very obvious oxidation traces on the surface of the 3D 4d SiC/SiC composite and the oxide layer peeling phenomenon was also observed after oxidation for 300 h. In Figure 12a, the defects of the sample were evenly distributed at the braided corners of the fiber bundles. After being oxidized, the defects near the surface were sealed with an increase in oxidation time (shown in Figure 12d,f,h). It can be observed from the area indicated by the orange arrow in Figure 12f that the defect pore directly connected to the outside was completely filled with oxide.

### 3.3. Mechanical Properties of Different Kinds of SiC/SiC

**Figure 13:**
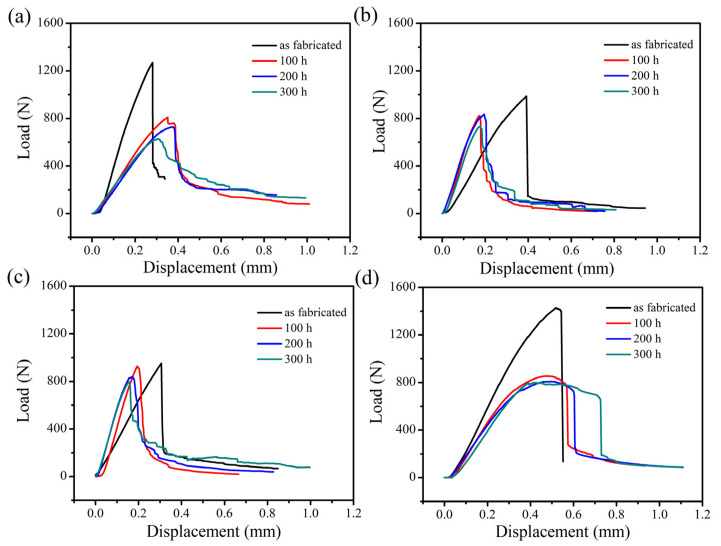
Load-displacement curves of four different preformed SiC/SiC composites without oxidation, 100 h, 200 h and 300 h after being oxidized: (**a**) 2D plain SiC/SiC; (**b**) 2D satin SiC/SiC; (**c**) 2D P/S SiC/SiC; (**d**) 3D 4d SiC/SiC.

**Figure 14:**
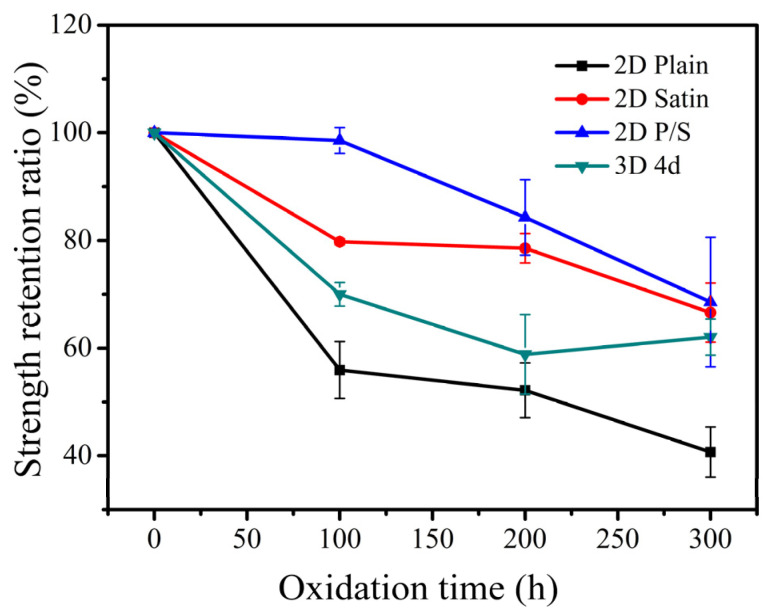
Strength retention rate of four SiC/SiC composites with different preforms after being oxidized for different times.

The three-point bending load-displacement curves of the four kinds of SiC/SiC composites at room temperature and the strength retention of the composites are exhibited in Figure 13 and Figure 14, respectively. According to the analysis of the graph, the fracture strength of the unoxidized composite was the highest and the flexural strength of the 2D plain, 2D satin and 3D 4d SiC/SiC showed a significant decrease when the materials were oxidized for 100 h. This effect demonstrated that the composites were seriously damaged under the enforcement of wet oxidation. Among them, the decrease of the bending strength of the 2D plain composite was the largest, which can be explained as follows: 1. The generated oxide accumulated at 0/90° fiber bundles braided holes due to the mismatch of the CTE (between the oxide and the composite), which could result in the formation of microcracks; 2. During the application of the three-point bending test, the microcracks would further expand, which ultimately led to a decline in the material bending strength. The 2D P/S composite still had a strength retention rate of about 98.57% after being oxidized for 100 h, illustrating that this structure, oxidized for 100 h, still maintained good mechanical properties. Interestingly, during the subsequent 200 h and 300 h stages of the oxidation, the four kinds of the composites did not show any significant decrease in flexural strength.

**Figure 15:**
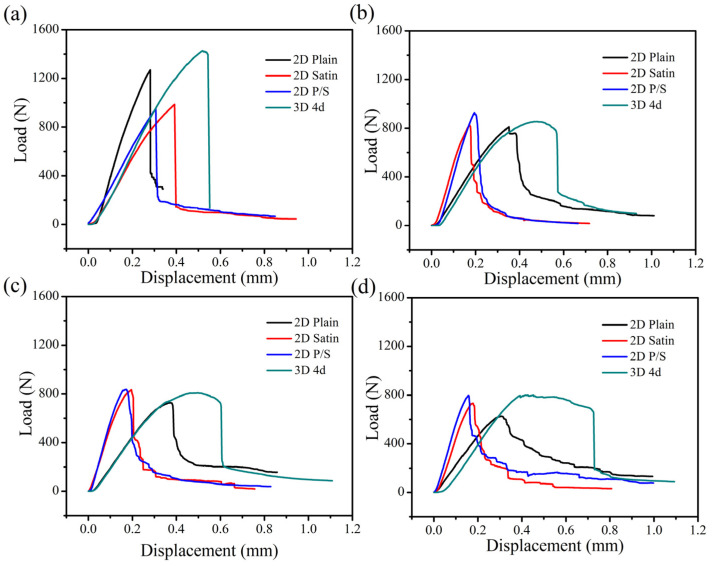
Load-displacement curves of SiC/SiC composites with four different preforms at different times: (**a**) as fabricated; (**b**) 100 h; (**c**) 200 h; (**d**) 300 h.

The comparison of the load-displacement curves of the four kinds of SiC/SiC composites with time is shown in Figure 15. A similar trend regarding the typical load-displacement curves of the composites can be found. Figure 15a represents the load-displacement curve of the as-fabricated composite material, suggesting that the mechanical strength of the composites decreased according to the following order: 3D 4d, 2D plain, 2D satin and 2D P/S composite. This signifies that the original 3D 4d SiC/SiC had the greatest flexural strength. Moreover, the four kinds of composites divulged a sharp drop in flexural strength after being oxidized for 100 h at 1400 °C, as can be seen from Figure 15b. Among them, although the flexural strength of the 3D 4d material was still the largest, the strength decreased by 80% compared with the as-fabricated samples. Interestingly, the oxidized 2D plain, 2D satin and 2D P/S composites reached the maximum breaking load, while the magnitude of the load drop decreased slowly (as is shown in Figure 15b–d). Furthermore, the changes of the 2D satin and P/S composites were closer, as can be observed. The oxidized 3D 4d composite also presented a sharp drop of 25–35% after reaching the maximum bending load, indicating that the proposed three-dimensional material retained some of the characteristics of the structure.

**Figure 16:**
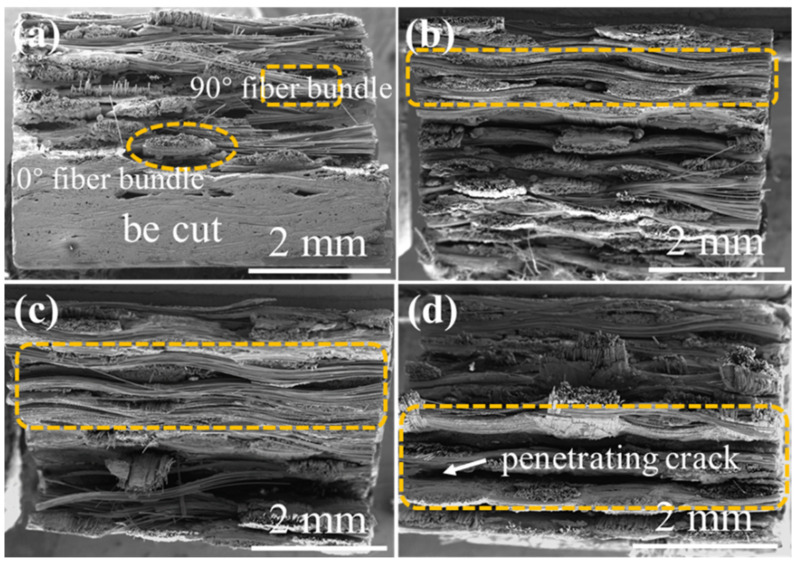
The fracture morphology of 2D plain SiC/SiC composites (**a**) before and after being oxidized at 1400 °C under the atmosphere of 12 kPa H_2_O:8 kPa O_2_:80 kPa Ar for (**b**) 100 h, (**c**) 200 h and (**d**) 300 h.

**Figure 17:**
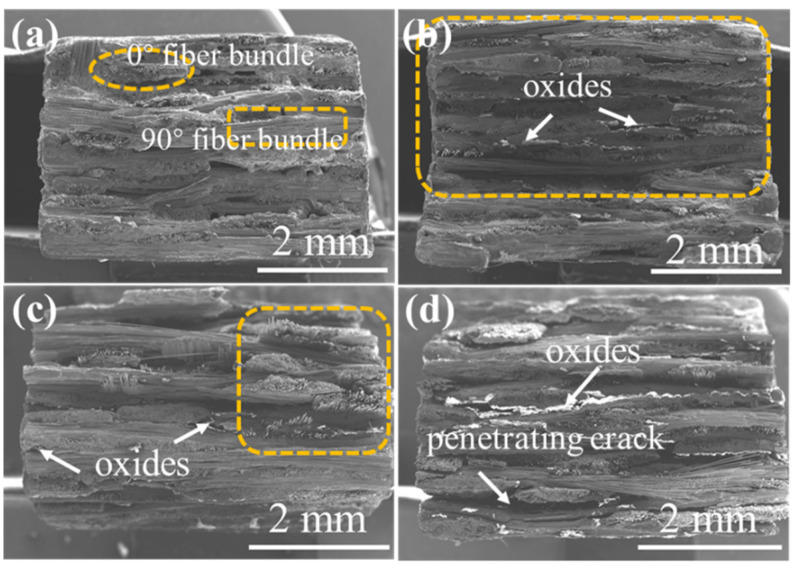
The fracture morphology of 2D satin SiC/SiC composites (**a**) before and after being oxidized at 1400 °C under the atmosphere of 12 kPa H_2_O:8 kPa O_2_:80 kPa Ar for (**b**) 100 h, (**c**) 200 h and (**d**) 300 h.

**Figure 18:**
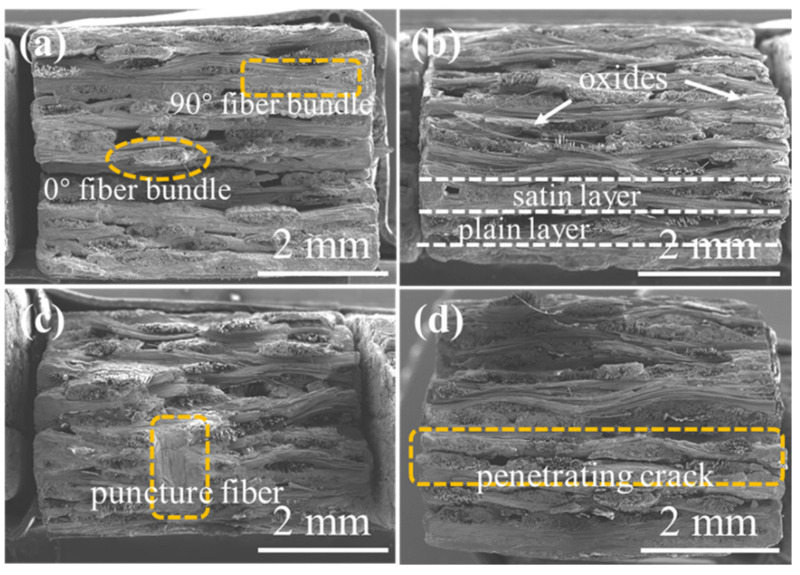
The fracture morphology of 2D P/S SiC/SiC composites (**a**) before and after being oxidized at 1400 °C under the atmosphere of 12 kPa H_2_O:8 kPa O_2_:80 kPa Ar for (**b**) 100 h, (**c**) 200 h and (**d**) 300 h.

**Figure 19:**
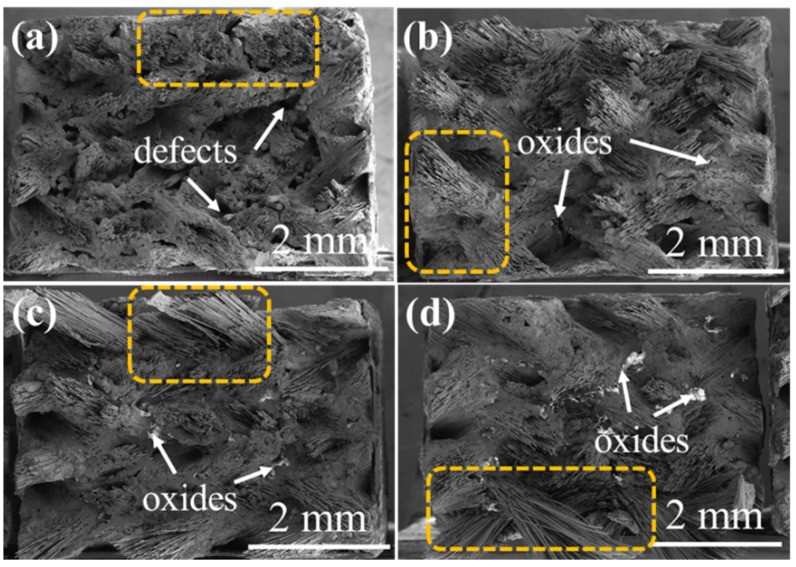
The fracture morphology of 3D 4d SiC/SiC composites (**a**) before and after being oxidized at 1400 °C under the atmosphere of 12 kPa H_2_O:8 kPa O_2_:80 kPa Ar for (**b**) 100 h, (**c**) 200 h and (**d**) 300 h.

The distribution and oxidation of the braided holes, the pull-out and oxidation of fibers, and the cracks propagation can be observed in Figure 16, Figure 17, Figure 18 and Figure 19, where the fracture morphologies of the four composite materials, under the application of 12 kPa H_2_O:8 kPa O_2_:80 kPa Ar atmosphere, at 1400 °C, are presented. Figure 16 displays the fracture morphology of the 2D plain SiC/SiC composite and the fracture morphology of the 2D plain weave composite material. Comparing the marked orange area in Figure 16, it can be clearly seen that the bond strength between the fiber cloth layers of the oxidized composite decreased. After being oxidized for 300 h (as shown in Figure 16d), the sample exhibited obvious penetrating cracks. Moreover, combined with the cross-sectional morphology of Figure 9, it is illustrated that the cracks generated during the oxidation process further propagated during the three-point bending test by transferring the inter-layers. The overall pull-out of fibers in the composite material were observed, and it was found that the pull-out length of fiber bundles gradually increased with the increase in oxidation time.

The fracture morphology of the 2D satin SiC/SiC composite is shown in Figure 17. In Figure 17b, oxides were formed at the braided pores between the fiber bundles, and only the single-filament fibers were pulled out. The overall fracture morphology was neater than that of the original sample shown in Figure 17a, indicating that oxidation played a role in strengthening the matrix, making the interlayer bonding strength stronger. When the sample was oxidized for 200 h (as shown in Figure 17c), the length of fiber pull-out was longer than that after oxidation for 100 h, and the bonding strength between layers also decreased. Besides, a penetrating crack appeared when the sample was oxidized for 300 h. It can be seen that the large crack at this time could be caused by the crack propagation during the three-point bending test combined with the cross-sectional morphology in Figure 10.

Figure 18 shows the 2D P/S SiC/SiC composite. It can be seen from Figure 18b that the defect holes were filled with oxides, and the plain layer and satin layer can be observed in the fracture morphology. When the sample was oxidized for 200 h (as shown in Figure 18c), a large area of obvious oxides could be found around the puncture fiber bundle, indicating that the presence of puncture fibers in the sample affected the oxidation behavior of the material. When the oxidation progressed to 300 h, it can be seen from the cross-sectional morphology, shown in Figure 11, that the cracks generated during the oxidation process were further transmitted along the layers under the action of the applied load.

As can be seen from Figure 19a, the defects existed at the angles between the fiber bundles. As the oxidation progressed, the oxides sealed the defects and the pull-out length of the fiber bundle gradually became longer with time (indicated by the orange area in Figure 19), which indicated that the damage at the interface increased, resulting in weakening of the bonding strength between the matrix and the fiber. In addition, it can also be found that the structural characteristics of the 3D 4d composites before and after oxidation were still obvious.

### 3.4. Theoretical Analysis of the μ-CT Test and Mass Change

The μ-CT was used to test the change of the volume fraction and distribution of defects within the sample. We have to underline that μ-CT is widely employed in the detection of microscopic systems of various materials, since it has the following advantages: 1. no specific requirement for sample preparation is necessary; 2. true non-destructive testing; 3. three-dimensional reconstruction. Therefore, μ-CT was selected as a means of detecting internal defects, including inter-bundle pores and intra-bundle pores of both the as-fabricated and the oxidized SiC/SiC composites. The underlying reason for choosing the defect volume fraction change rate as the starting point is that the macroscopic weight change of the material is reflected in the internal defect change of the sample. Consequently, by constructing the function between the defect volume fraction change rate and the mass change rate, the quantitative relationship between the micro- and the macro-system can be explained.

The defect volume fraction change rate and the mass change rate are also respectively used as the dependent variable and independent variable to develop the following functions:

(1) $$D=\frac{v_{d}-v_{0}}{v_{0}}\times 100\%$$

(2) $$W=\frac{m-m_{0}}{m_{0}}\times 100\%$$

where *D* in Equation (1) is the rate of change of defect volume, and *v_0_* and *v_d_* represent the defect volume fraction of the original and the oxidized composites, respectively. W stands for the mass change rate, *m* and *m*_0_ are the weight of as-fabricated and the oxidized materials.

### 3.5. Function between the Defect Volume Change Rate and Mass Change Rate

**Figure 20:**
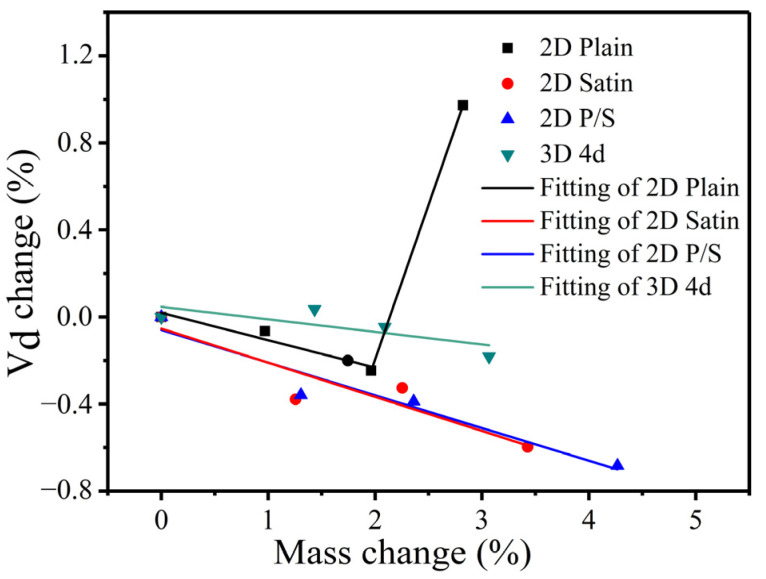
Fitting function of defect volume fraction change rate and mass change rate of four kinds of preform structure composite.

The fitting function relationship between the change rate of the internal defect volume fraction and the mass change rate of the four kinds of composites is displayed in Figure 20. As can be ascertained, only the 2D plain composite had increased after being oxidized for 200 h. This effect is mainly ascribed to the 2D plain SiC/SiC composite having more oxidation products formed at the weaving holes of the fiber bundles in the late oxidation stage. In addition, the CTE mismatch issue between the oxides and composites arose, which led to the manifestation of an obvious delamination effect between the layers. Hence, the defect volume fraction increased significantly when the μ-CT scanning was performed. Under this direction, the functional relations constructed by the four kinds of composite materials are described as follows.

2D plain SiC/SiC:

(3) $$D_{1}=\{\begin{array}{c} -0.12589\times W+0.01921\;\;\;\;\;\;\;\;\;0<t\leq 200\\ \;\;1.4186\times W-3.03131\;\;\;\;\;\;\;\;\;\;200<t\leq 300 \end{array}$$

2D satin SiC/SiC:

(4) $$D_{2}=-0.1567\times W-0.0532\;\;\;\;\;\;\;\;0<t\leq 300$$

2D P/S SiC/SiC:

(5) $$D_{3}=-0.1515\times W-0.05954\;\;\;\;\;\;\;\;0<t\leq 300$$

3D 4d SiC/SiC:

(6) $$D_{4}=-0.05761\times W+0.04676\;\;\;\;\;\;\;\;0<t\leq 300$$

In the above-mentioned function, *Di* (*i* =1, 2, 3, 4) corresponds to the change rate of the defect volume fraction of the four different kinds of composites, and *t* = 100, 200, 300 (h), represent the different oxidation times.

By observing the functions of the four kinds of composites, it can be found that the slopes of the straight lines of the 2D satin and the 2D P/S composites are similar and followed by the 2D plain and 3D 4d composites (decreasing in order). This effect indicates that the defect volume change rate of the 2D satin and 2D P/S composites changed fast as the mass change rate became bigger. The above four formulas are recorded as *D* = *AW* + *B* (*A* and *B* are both constants), which can be further simplified as *m* = *m*_0_ + *p* × *m*_0_, *p* = (D−B)/A. It can also be acquired, from the simplified expression, that if the initial mass of the composite material is known, the weight of the sample after performing oxidation for different times can be calculated. Finally, it is proved that the proposed method is feasible, and the calculated results are close to the experimental data.

## 4. Conclusions

The CVI method was used to deposit the interface and matrix of the composites with four different preforms. Finally, SiC/SiC composites with an open porosity of 9% were prepared. After the samples were oxidized under an atmosphere with the partial pressure of 12 kPa H_2_O:8 kPa O_2_:80 kPa Ar, at 1400 °C, a series of characterizations could be performed to obtain the following conclusions:

1. Although the mass change rate of the four composites increased with time, the oxidation rate gradually slowed down. The mass change rate of 2D P/S SiC/SiC was the largest among the four composite materials with different preforms. According to the XRD results and micro-morphology analysis, it can be seen that SiO_2_ generated on the surface can slow down the diffusion of the oxidative atmosphere and heal surface cracks. The oxides generated inside the material played the role of filling defects. The oxidizing atmosphere diffused from the outside to the inside of the composites. The interlayer oxidation of the three two-dimensional materials was faster than that of the cross-section direction, while the 3D 4d composites displayed uniform oxidation.
2. A three-point bending strength test was performed on the original and oxidized samples, and it was found that the bending strength of the four composites decreased significantly, which demonstrates that the materials were damaged to varying degrees after oxidation. However, it is worth mentioning that the flexural strength of the 2D P/S composite reached 98% when oxidized for 100 h, which indicates that the material has good mechanical properties in the early stage of oxidation.
3. Micro-computed X-ray tomography (μ-CT) measurement is an effective method to obtain the defect distribution of composites. The function between the defect volume fraction change rate and the mass change rate was constructed, based on the non-destructive testing method. The qualitative and quantitative relationship between the microstructure and the macroscopic properties was established to realize the evaluation of the oxidation behavior of composite materials.

## Figures and Tables

**Table 1:** Properties of the SiC fiber.

Diameter (μm)	Density (g/cm^3^)	Tensile Strength (GPa)	Tensile Modulus (GPa)	Strain of Failure (%)
14.0	3.0	3.5	369.8	0.9

## Data Availability

The data presented in this study are available on request from the corresponding authors.
